# A Novel Method to Combine Maxilla-Based Coordinate System and Mandibular Voxel-Based Superimposition with Cone-Bean Computed Tomography

**DOI:** 10.3390/jcm11175229

**Published:** 2022-09-04

**Authors:** Chenghao Zhang, Ling Ji, Yijun Li, Fangwei Pan, Wen Liao, Zhihe Zhao

**Affiliations:** 1 State Key Laboratory of Oral Diseases & National Clinical Research Center for Oral Diseases, Department of Orthodontics, West China Hospital of Stomatology, Sichuan University, Chengdu 610041, China; 2 State Key Laboratory of Oral Diseases & National Clinical Research Center for Oral Diseases, West China Hospital of Stomatology, Sichuan University, Chengdu 610041, China; 3 State Key Laboratory of Oral Diseases & National Clinical Research Center for Oral Diseases, Department of Prosthodontics, West China Hospital of Stomatology, Sichuan University, Chengdu 610041, China

**Keywords:** CBCT, three-dimensional evaluation, voxel-based superimposition, coordinate system

## Abstract

Background: The objective of this study was to propose a method that combines a maxilla-based coordinate system and mandibular voxel-based superimposition for an accurate evaluation of mandibular structural and positional changes and a direct comparison between maxillary and mandibular structural changes with the same 3D vectors. Methods: Mandibular voxel-based superimposition was firstly performed to reorient the mandibles and eliminate the mandibular positional changes. Then, a maxilla-based coordinate system was constructed with four maxillary skeletal landmarks (ANS, PNS, OrL and OrR). After settling the reoriented mandibles into this coordinate system, the mandibular structural changes were accurately evaluated. To assess the accuracy and reproducibility of this method, CBCT images of a skull specimen before and after orthodontic treatment (which was simulated by rearranging the skull and the mandible) were collected. Five mandibular skeletal landmarks, three mandibular dental landmarks and two mandibular measurement planes of this skull were used to evaluate the linear and angular changes in the mandibular structures. Results: There were significant differences in the linear and angular measurements of the mandibular structures of the skull (*p* ˂ 0.05), which indicated mandibular positional changes after orthodontic treatment. After mandibular voxel-based superimposition, there were no significant differences in the linear and angular measurements of mandibular structures, which indicated that the mandibular positional changes were eliminated. The intraclass correlation coefficient (ICC) value of the inter- and intra-observer agreement of all measurements was 0.99. Conclusions: This method has proven advantages in terms of accuracy, reproducibility and validity; with this method, mandibular structural and positional changes can be accurately evaluated and maxillary and mandibular structural changes can be directly compared with same 3D vectors.

## 1. Introduction

Cone-beam computer tomography (CBCT) is an increasingly widely used radiological technology in orthodontics because of its accurate three-dimensional (3D) performance, low distortion rate and relatively low radiation [1,2]. Three-dimensional reconstruction and superimposition make it easy to accurately evaluate treatment effects and analyze the skeletal and dental changes before and after treatment [3]. For measuring and comparing the three-dimensional positions of skeletal and dental structures, the common method is to set coordinate systems in the two 3D skull models reconstructed, respectively, with pre- and post- treatment CBCT images. In this process, most of the coordinate systems use maxillary landmarks as reference points, because these landmarks are relatively stable throughout treatment [4,5,6]. Our previous study has also proposed a maxilla-based personalized coordinate system, which is more user-friendly and can accurately evaluate the position changes in maxillary teeth and alveolar bones before and after orthodontic treatment [7]. However, none of the coordinate systems using maxillary landmarks is applicable for an accurate evaluation of the mandibular structural changes or the maxilla–mandible relationship in cases of functional orthopedic treatment or orthognathic surgery. The reason is that the mandible is the only moveable bone of the skull [8,9]. As a process of occlusal reconstruction, orthodontic treatment may cause changes in the spatial position of the mandible [10,11,12,13], which normally manifest as angular changes in the occlusal plane and mandibular plane. Both structural changes and positional changes in the mandible caused by occlusal change are recorded in this coordinate system, which leads to unreliable results. To eliminate the interference effects of mandibular positional changes on the mandibular structural changes, our solution is to superimpose the mandibles before and after treatment and place them in the same coordinate system. Although a coordinate system using only mandibular landmarks may solve this problem [14], this solution also has its own disadvantage, i.e., it is hard to find enough mandibular skeletal landmarks that are stable and easy to locate to construct a mandible-based coordinate system. Additionally, no matter whether using a maxilla- or mandible-based coordinate system, none of these methods could evaluate the maxillary and mandibular structural changes in a same coordinate system or provide a direct comparison between these changes. Thus, it is necessary to find out a novel method that could be exempted from the above-mentioned disadvantages of the current methods.

In recent years, voxel-based superimposition, a technique that automatically matches the voxel grayscale values of CBCT volumes [15], has drawn the attention of orthodontic clinicians. It is considered promising by virtue of its good validity and reliability, and high repeatability, as well as for being less time-consuming [16,17]. With voxel-based superimposition, the mandibles before and after treatment could be easily superimposed, and the changes in specific mandibular structures could be clearly exhibited, since the pseudo changes caused by mandibular positional changes are eliminated [18]. However, superimposition alone could only evaluate the changes in certain structures, which is largely a qualitative measurement, with insufficient 3D quantitative measurement and analysis. In orthodontics, establishing proper 3D vectors as reference standards is necessary to understand the structural changes caused by three-dimensional force components, and such understanding is helpful for guiding treatment decision making. Hence, a stable and reliable coordinate system is still needed.

The aim of this study is to propose a method that combines a maxilla-based coordinate system and mandibular voxel-based superimposition to accurately evaluate mandibular structural changes. It is also a potential method to evaluate and compare the maxillary and mandibular structural changes in a same coordinate system. With its proven advantages in terms of accuracy, reproducibility and validity, this method can be applied in orthodontics to measure the three-dimensional effects of treatment that may involve mandibular positional changes, such as functional-appliance treatment and orthognathic surgical treatment, as well as in prosthodontics, dental implantology, and oral and maxillofacial surgery treatment analyses.

## 2. Materials and Methods

### 2.1. Research Object and Data Collection

**Figure 1:**
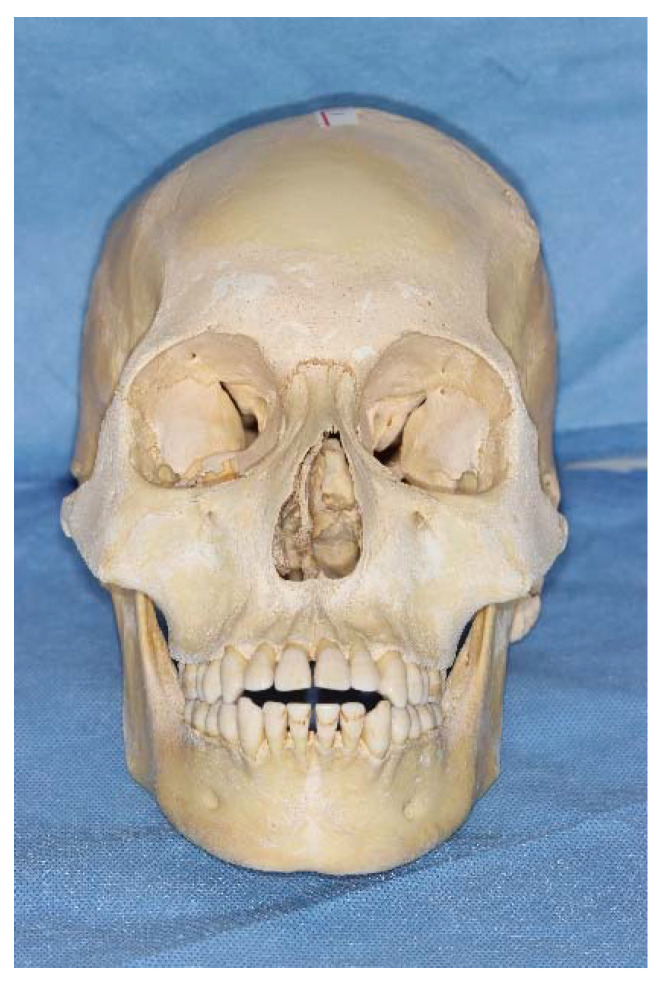
A human skull specimen was selected as the research object.

A human skull specimen from Department of Dental Anatomy, West China College of Stomatology, Sichuan University, was selected as the research object (Figure 1). It had a normal skeleton, all teeth from the central incisors to the second molars and repeatable maximum intercuspal position (ICP). As the research object, its mandible was separated from the skull, which allowed us to manually rearrange the mandibular position. The skull was set at its ICP, and the CBCT images of the skull were firstly obtained (T0). Then, this skull and its mandible were randomly placed to simulate different head positions when taking CBCT images and different mandibular position after orthodontic or orthognathic treatment. The CBCT images were taken again (T1). All the CBCT images were taken with the same CBCT machine (3D Accuitomo; Morita Group, Japan), which was set according to the manufacturers’ recommendations (140 × 100 mm FOV, 85 kV, 4.0 mA and 360° rotation). The voxel size was 125 μm. The CBCT data were stored in DICOM multifile format.

Before the measurements, the DICOM data at both T0 and T1 were imported into Dolphin software (Version 11.8; Dolphin Imaging & Management Solutions, Chatsworth, CA, USA), and the head positions were three-dimensionally reoriented with the Frankfort plane parallel to the ground.

### 2.2. Mandibular Voxel-Based Superimposition

**Figure 2:**
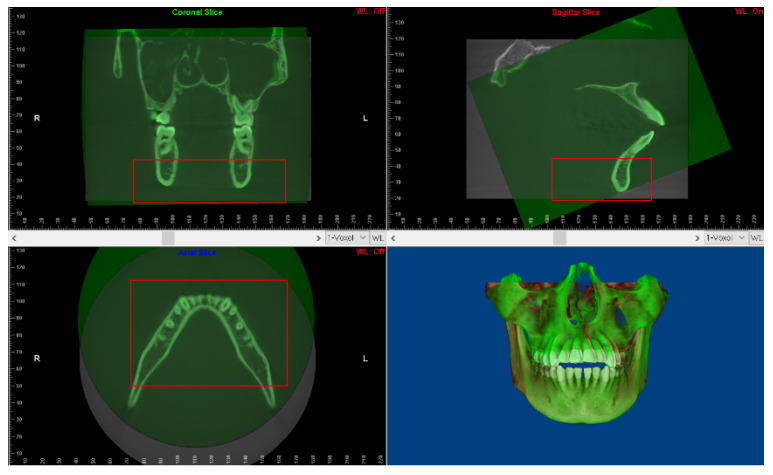
The automatic voxel-based superimposition using the registration reference area of the basal bone of the mandibular body containing no teeth nor alveolar bones.

**Figure 3:**
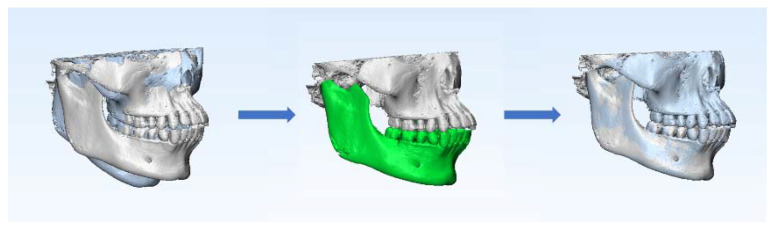
Process of mandibular voxel-based superimposition between T0 and T1. The white skull is the 3D model at T0. The blue skull is the 3D model at T1. The green area is the registration reference area of mandibular voxel-based superimposition.

According to the voxel-based superimposition protocols [19], the data at T0 were set as the base volume in Dolphin software, and the data at T1 were set as the second volume. The superimposition of the two volumes involved the following three steps: Its first step was landmark-based superimposition; four landmarks were selected as reference points, namely, the most mesial points of the left and right mental foramina, and the deepest points of the left and right antegonial notches. The next step was automatic voxel-based superimposition using the registration reference area of the basal bone of the mandibular body containing no teeth or alveolar bones [20,21] (Figure 2). After that, the two volumes were automatically superimposed; meanwhile, the head orientation of the second volume was altered in accordance with the 3D position of the base volume (Figure 3). The final step was to export the second volume in DICOM multifile format, which was defined as the T2 volume.

### 2.3. Construction of Maxilla-Based Coordinate System and Location of Mandibular Measurement Landmarks

The DICOM data at T0, T1 and T2 were then respectively imported into Mimics Research software (Version 19.0; Materialise, Leuven, Belgium). The optimal gray-value range of bone tissue in each CBCT slice was considered the threshold and was segmented. After that, the 3D models were reconstructed.

**Figure 4:**
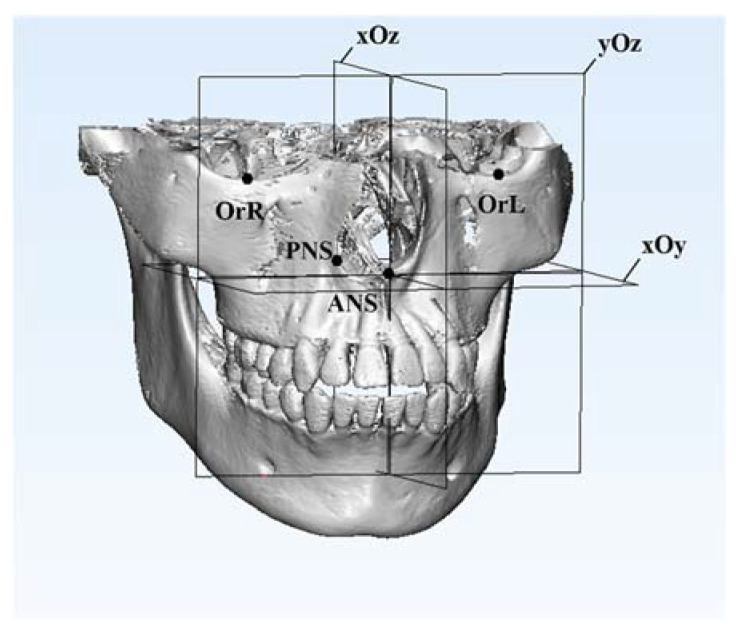
The construction of the maxilla-based coordinate system was based on four basic landmarks: ANS, PNS, OrL and OrR. The ANS point was defined as the origin of the coordinates. The black points are these four basic maxillary landmarks.

According to our former research, four maxillary landmarks (ANS, PNS, OrL and OrR) were selected as the basic coordinates to construct the maxilla-based coordinate system in the T0 and T1 models. The ANS point was defined as the origin of the coordinates. The horizontal plane (xOy) was defined as the plane passing through ANS and PNS, while parallel to the line OrL-OrR. The sagittal plane (yOz) was defined as the plane passing through ANS and PNS while perpendicular to the horizontal plane. The frontal plane (xOz) was defined as the plane passing through ANS while perpendicular to both the horizontal plane and the sagittal plane. (Table 1 and Figure 4.)

**Figure 5:**
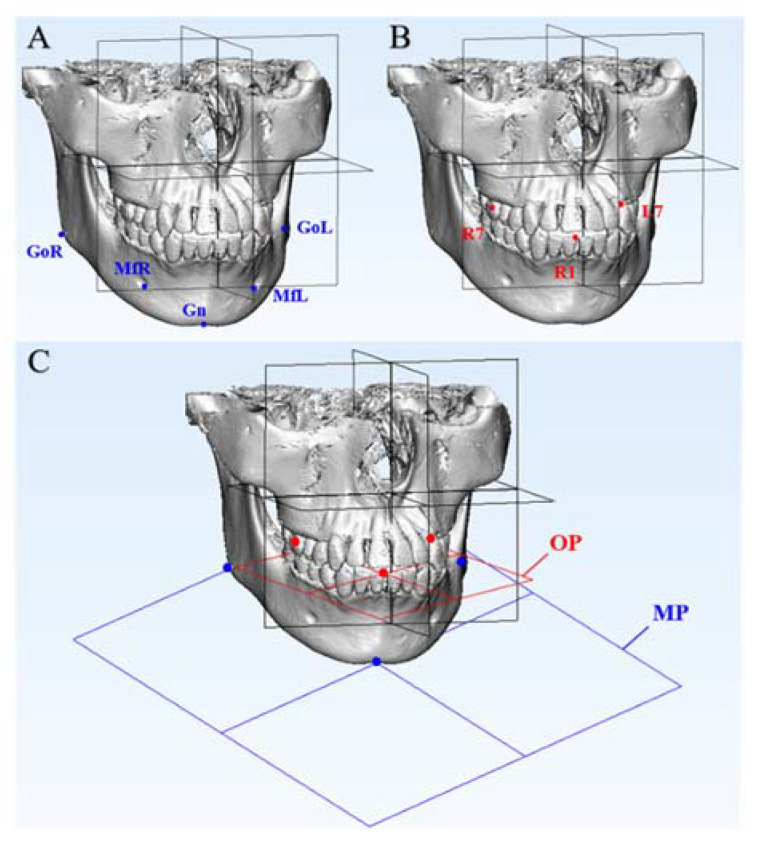
Mandibular measurement landmarks and planes. (**A**) The blue points are five mandibular skeletal landmarks: MfL, MfR, GoL, GoR and Gn. (**B**) The red points are three mandibular dental landmarks: R1, L7 and R7. (**C**) The blue plane is the mandibular plane constructed by Gn, GoL and GoR. The red plane is the occlusal plane constructed by R1, L7 and R7.

Five mandibular skeletal landmarks, three mandibular dental landmarks and two mandibular measurement planes were used to evaluate the linear and angular changes in the mandibular structures in the 3D models at T0, T1 and T2, respectively, as shown in Table 2 and Figure 5. The locating of all the maxillary and mandibular landmarks was based on sagittal, coronal and transversal slices of CBCT images and 3D models.

**Figure 6:**
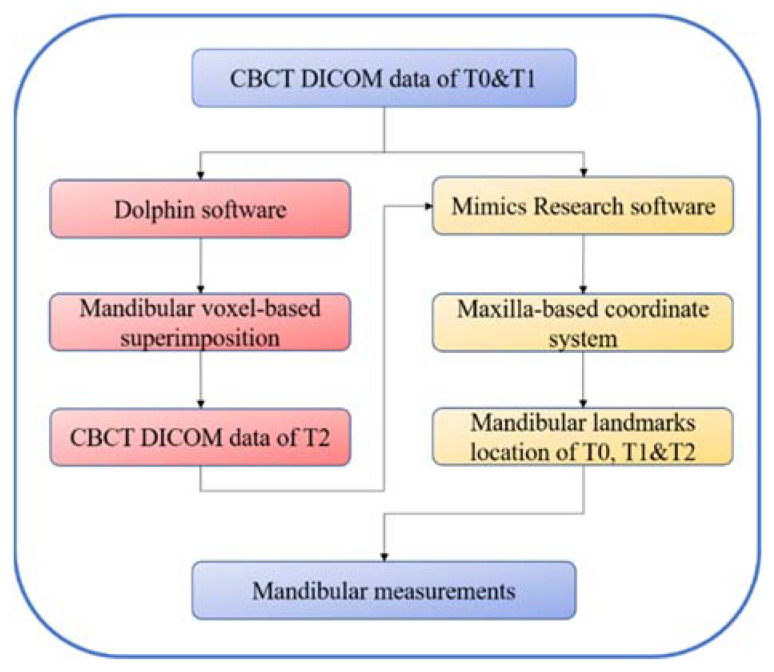
Schematic diagram of the process of this method. T0, before treatment; T1, after treatment, which was simulated via mandibular rearrangement; T2, after mandibular voxel-based superimposition on T1 CBCT data.

The schematic diagram of the process of this method is shown in Figure 6.

### 2.4. Data Analysis and Statistics

Firstly, the linear dimensions of maxillary basic landmarks at T0 and T1 were evaluated repeatedly to verify the accuracy and reproducibility of the maxilla-based coordinate system. The distances from ANS to PNS, from OrL to OrR, and from OrL and OrR to the xOy, xOz and yOz planes were measured.

Then, the differences in the linear and angular dimensions of the mandibular landmarks at T0 and T1 were measured. These three-dimensional differences represented the total changes after orthodontic treatment, including mandibular structural and positional changes, as simulated via the manual rearrangement of the skull and the mandible.

Next, the T2 mandible was transferred into the T0 coordinate system so that the mandibular positional changes caused by occlusal change could be eliminated. The differences in the linear and angular dimensions of the mandibular landmarks at T0 and T2 and in those at T1 and T2 were measured.

In our research study, the mandibular differences between T0 and T1 represented the total changes in the mandible, including the mandibular structural changes and positional changes. After mandibular voxel-based superimposition, the mandible at T1 was reoriented to the T2 position. Then, the differences between T0 and T2 represented the mandibular structural changes, and the differences between T1 and T2 represented the mandibular positional changes. The relationships of the differences among T0, T1 and T2 can be summarized with the following formulas:

Diff. (T0 & T1) = mandibular total changes

Diff. (T0 & T2) = mandibular structural changes

Diff. (T1 & T2) = mandibular positional changes

Diff. (T0 & T1) = Diff. (T0 & T2) + Diff. (T1 & T2)

All the operations and measurements were conducted three times each and independently by two operators under identical conditions. Statistical evaluations were performed with SPSS software (Version 22.0; IBM, Armonk, NY, USA). A paired *t*-test was used to evaluate the differences in the linear dimensions of the maxillary basic landmarks at T0 and T1. One-way repeated measures ANOVA and Tukey’s multiple comparisons test were used to evaluate the linear and angular changes in the mandibular structures at T0, T1 and T2. The Bland–Altman plot analysis was used for investigating the inter-observer agreement in the measurements. The intraclass correlation coefficient (ICC) was used to assess the inter- and intra-observer agreement. The threshold of statistical significance was set at 0.05.

## 3. Results

**Figure 7:**
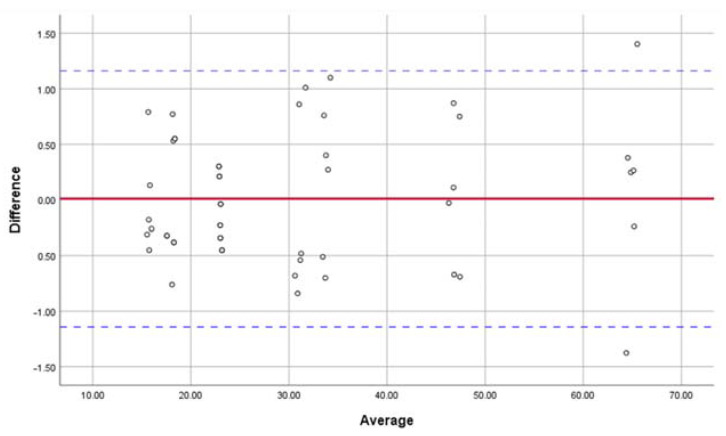
Bland–Altman plot analysis was used for investigating the inter-observer agreement in locating the maxillary basic landmarks and measuring the linear dimensions, which indicated great reproducibility in constructing the maxilla-based coordinate system.

The linear dimensions of the maxillary basic landmarks at T0 and T1 were evaluated, and no significant differences were found (Table 3). The SDs of the measurements were all <0.7 mm, and the ICC values of the inter- and intra-observer agreement for the linear maxillary measurements were both 0.99. The results indicated great precision and reproducibility of the construction of the maxilla-based coordinate system. The Bland–Altman plot analysis was used for investigating the inter-observer agreement, which also indicated great reproducibility (Figure 7).

**Figure 8:**
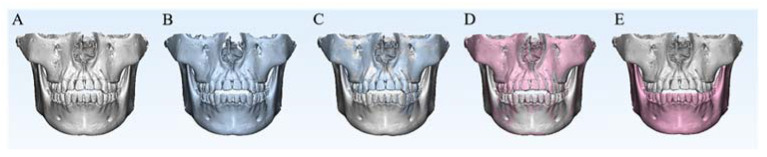
The position changes among T0, T1 and T2. (**A**) The white skull is the 3D model at T0. (**B**) The blue skull is the 3D model at T1. (**C**) Differences in the spatial positions of the mandible between T0 and T1, which included mandibular structural changes and mandibular positional changes caused by occlusal change. (**D**) The red skull is the 3D model at T2. After mandibular voxel-based superimposition, the positional changes between T0 and T1 were transferred to the maxilla, and there were no differences in the spatial positions of the mandible between T0 and T2. (**E**) After placing the mandible at T2 in the T0 maxilla coordinate system, the positional changes caused by occlusal change were eliminated, and the mandibular structural changes could be evaluated.

The linear and angular changes in the mandibular structures were measured. Comparing T0 and T1, there were increases in the distances from six out of eight mandibular landmarks to the frontal plane, which indicated the backward movement of the mandible. There were increases in the distances from all three left mandibular landmarks (L7, MfL and GoL) to the sagittal plane, while the distances from two out of three right mandibular landmarks (R7 and MfR) decreased, which indicated that the mandible deviated to the left. There were no significant differences in the positional changes in GoR. One possible reason was that the rotation center of the mandible was near GoR. The mandibular structural changes are shown in Figure 8A–C.

After mandibular voxel-based superimposition, the mandibular positional changes between T0 and T1 were transferred to the maxilla, and there were no differences in the spatial positions of the mandible between T0 and T2. After placing the mandible at T2 in the T0 maxilla coordinate system, the positional changes caused by occlusal change were eliminated, and the mandibular structural changes could be evaluated (Figure 8D–E).

**Figure 9:**
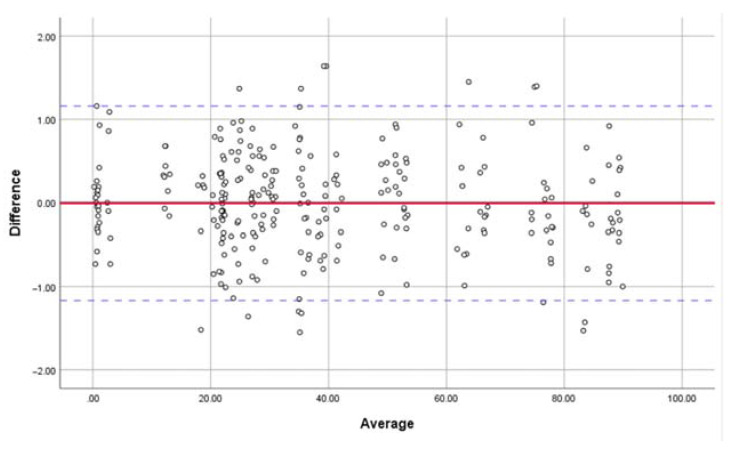
The Bland–Altman plot analysis was used for investigating the inter-observer agreement in locating the mandibular measurement landmarks and measuring the linear dimensions, which indicated great reproducibility.

In this study, the mandibular structures of the skull were stable, so there were no significant differences in the linear and angular measurements at T0 and T2. The differences between T1 and T2, which represented the mandibular positional changes, were mostly similar to the differences between T0 and T1 (Table 4, Table 5 and Table 6). The SDs of all the linear measurements were <1.0 mm, and the SDs of all the angular measurements were <0.8°. The ICC values of the inter- and intra-observer agreement for linear and angular mandibular measurements were both 0.99. The Bland–Altman plot analysis also indicated its great reproducibility (Figure 9).

## 4. Discussion

Our study aimed to propose a novel method to evaluate the three-dimensional mandibular structural changes. Its accuracy and reproducibility are experimentally supported by our linear and angular measurements of a skull’s mandibular structures. For simulating the orthodontic treatment, CBCT images of a skull taken pretreatment (T0) and post-treatment (T1) were collected. Then, a maxilla-based coordinate system was constructed with four maxillary basic landmarks: ANS, PNS, OrL and OrR. They were selected for their relative stability and for being easy to locate; moreover, they generally make the coordinate system clinician-friendly, as they are pervasively used in cephalometric analyses in orthodontics. ANS was defined as the origin of the coordinate system, and the line through OrL-OrR was used as a guideline to help set up the basic plane, namely, the horizontal plane (xOy) that passes through ANS and PNS while being parallel to the line OrL-OrR. After the horizontal plane was set up, the sagittal plane (yOz) and the frontal plane (xOz) could be defined. In this way, the Euclidean coordinate system was successfully constructed. In theory, this maxilla-based coordinate system could be set up for every case based on their CBCT data. Even in an orthognathic surgery case with maxillary surgery where the point of ANS may change, the cranial base could be set as reference area for maxillary superimposition so that the coordinate system at T0 is still valid and stable for measurement.

Followed by mandibular voxel-based superimposition, the differences in the mandibular structural measurements were compared among T0, T1, and T2. These differences indicated that the mandible deviated to the back side and left side, while no structural changes were observed during orthodontic treatment, which was in accordance with the manual rearrangement of the skull and the mandible.

This method is innovative in how it combines mandibular voxel-based superimposition and a maxilla-based coordinate system to successfully eliminate the pseudo positional changes caused by occlusal change and achieve precision in measuring mandibular structural changes. As a process of occlusal reconstruction, orthodontic treatment may cause changes in the spatial position of the mandible, especially in cases of functional-appliance treatment, splint therapy and orthognathic surgery [26,27,28]. Additionally, condylar position may change due to multiple factors, including ages, disc displacement and teeth extraction [29,30,31,32]. To eliminate the interference effects of mandibular positional changes on the mandibular structural changes, our solution was to superimpose two mandibles, representing the before- and after-treatment stages, and place them in the same coordinate system to calculate and compare the measurements of the mandibular structures. With this method, the mandibular structural changes could be easily calculated by comparing the mandibles at T0 and T2 in the same maxilla-based coordinate system. Meanwhile, mandibular positional changes could also be evaluated using this method. This is meaningful for functional orthopedic treatment and orthognathic surgery, in which cases the changes in mandibular morphology make it hard to distinguish between and evaluate the mandibular positional changes and structural changes. In this study, because the skull was stable, there should have been no differences between T0 and T2; hence, the differences between T0 and T1 should have been equal to the differences between T1 and T2. This was supported by our measurements.

Another innovative advantage of this method is that it allows researchers to directly evaluate and compare the structural changes in the maxilla and mandible in a same coordinate system, which further means they could share the same 3D vectors. This is potentially beneficial for some clinical research studies. For example, considering that the force of Class II elastics would affect both maxillary and mandibular dentition, to measure and analyze the changes in maxillary and mandibular dentitions with the same reference standard could be helpful for understanding the three-dimensional force components of Class II elastics and their effects. This advantage is meaningful for functional orthopedic treatment and orthognathic surgery. The method remains applicable even for some cases where growth factors or orthopedic surgery may cause maxillary structural changes and interfere with the construction of the T1 maxilla. Theoretically, in these cases, the T0 coordinate system is still set as the basic coordinate system; the maxillary voxel-based superimposition at T1 is conducted using the anterior cranial base as reference [33,34]; the combination with the mandibular voxel-based superimposition we propose is conducted; then, maxillary and mandibular structural changes can be directly compared with the same reference standard. Besides its usefulness for clinical research, the unification of the 3D vectors between the maxilla and mandible is potentially conductive for the digital industrial manufacturing of oral medicine.

Recently, most of the research studies in orthodontics utilize CBCT images for three-dimensional measurements for their good reliability, as well as in prosthodontics, dental implantology, and oral and maxillofacial surgery [35,36]. However, there is no standard method for their measurement. There are three primary methods. First, some researchers directly locate the anatomic skeletal and dental landmarks on CBCT images with proper view sections to measure the linear dimensions between these landmarks [37,38,39,40]. However, this method can only provide linear measurements between certain landmarks but not a three-dimensional treatment assessment of treatment effects on dental and skeletal structures. Additionally, the reproducibility and accuracy of this method may not be good enough considering that the precision of the results is influenced by whether the chosen view sections are proper.

The second method is to set up a coordinate system that can provide 3D vectors for three-dimensional measurements [41,42,43]. Most of the coordinate systems are based on maxillary landmarks or planes. Under the interference effects of mandibular positional changes, these systems cannot provide an accurate assessment of mandibular structural changes. The rest of the coordinate systems are constructed with mandibular skeletal landmarks [14]. Although it would not be interfered with by mandibular positional changes, this type of system is restricted to measuring mandibular structural changes. In other words, there is no single coordinate system that can measure mandibular structural changes without interference effects while also measuring maxillary structural changes at the same time. To create such system is needed, as it would enable us to directly compare the maxillary and mandibular structural changes with the same 3D vectors.

With the third method, some researchers superimpose the maxillary and mandibular structures before and after treatment to exhibit and evaluate their changes [44,45,46]. They reconstruct the 3D models and then use the landmark-based, surface-based or voxel-based method to superimpose the models and compare their differences. Unfortunately, superimposition alone normally uses color maps to compare the structural changes, and it provides primarily qualitative data but insufficient 3D quantitative analysis. As it cannot decompose and express the differences in the three dimensions with proper vectors as reference standards, it is unsatisfactory in reaching a comprehensive understanding of three-dimensional treatment effects. Although some measurement software could provide a 3D vector with its equipped common coordinate system, researchers would still have to conduct an additional step to manually reorient the head position [18,47], which is time-consuming. In addition, it is less researcher-friendly in that the accuracy of its results is overtly dependent on the great precision of manual head reorientation. Additionally, when it comes to measuring the structural changes and comparing those changes between different cases, it could be uneasy to use and to give sufficiently clear results, as software coordinate systems has no origin of coordinates and actual coordinate planes.

This study had some limitations. Firstly, only one dry human skull was selected; two CBCT records were obtained; and measurements were repeated for analysis six times. The reason was that the purpose of this study was to explore a possible method for clinical research. Using one dry skull as the research object could avoid the interference of confounding factors in the study. Secondly, the analysis of the changes occurring during treatment was based on a simulation, and the accuracy and validity of this method were theoretically proved by previous works but not fully validated. Therefore, we remind readers that in clinical settings, relevant interference is usually ineluctable and that the results of this study should be treated with great caution. In our future work, we aim to expand the samples’ quantity and focus on practical clinical situations.

Taking all the above into consideration, the proposed method has several advantages. It is time-saving and user-friendly and shows great accuracy, reproducibility and validity. The radiation dose of its needed CBCT images could be relatively low; the imaging field of its CBCT images is only expected to cover the cranial and maxillofacial skeletal structures from the orbitales to the mandibular body; hence, the medium field of view would be enough [48,49]. Moreover, theoretically, there would be no need for precise head orientation because the coordinate system is constructed with certain maxillary basic landmarks and has great reproducibility. This method could be helpful for clinical research that involves the description of the mandibular structural and positional changes and the comparison between maxillary and mandibular structural changes. It can be applied in orthodontics to assess three-dimensional treatment effects, as well as in prosthodontics, dental implantology, and oral and maxillofacial surgery treatment analyses.

## 5. Conclusions

A systematic method that combines a maxilla-based coordinate system and mandibular voxel-based superimposition is proposed in this study. It not only enables us to accurately evaluate the mandibular structural and positional changes, but it can also be used to directly compare the maxillary and mandibular structural changes with the same reference standard.

## Figures and Tables

**Table 1:** Maxillary basic landmarks and planes of the maxilla-based coordinate system.

Maxillary Basic Landmarks and Basic Planes	
OrL	The most inferior point of the left bony orbit
OrR	The most inferior point of the left bony orbit
ANS	The tip of the anterior nasal spine
PNS	The tip of the posterior nasal spine
xOy	The horizontal plane, passing through ANS and PNS while parallel to the line OrL-OrR
yOz	The sagittal plane, passing through ANS and PNS while perpendicular to the horizontal plane
xOz	The frontal plane, passing through ANS while perpendicular to both horizontal plane and sagittal plane

The definition and location of the maxillary landmarks and planes were based on previous research studies [22,23,24].

**Table 2:** Mandibular measurement landmarks and planes.

Mandibular Measurement Landmarks and Planes	
MfL	The most anterior point of the left mental foramen
MfR	The most anterior point of the right mental foramen
GoL	The midpoint of the bony border of the left mandibular angle
GoR	The midpoint of the bony border of the right mandibular angle
Gn	The intersection between the mid-sagittal plane and the most anteroinferior point of
	the mandibular symphysis
R1	The midpoint of the incisor edge of the mandibular right incisor
R7	The mesial–buccal cusp tip of the mandibular right second molar
L7	The mesial–buccal cusp tip of the mandibular left second molar
OP	Occlusal plane, constructed by R1, L7 and R7
MP	Mandibular plane, constructed by Gn, GoL and GoR

The definition and location of the mandibular landmarks and planes were based on previous research studies [14,23,24,25].

**Table 3:** The linear dimensions of basic maxillary landmarks at T0 and T1.

	T0		T1		*p*-Value
	Mean (mm)	SD	Mean (mm)	SD	
ANS-PNS	46.8	0.35	47.04	0.66	0.4496
OrL-OrR	64.96	0.64	64.9	0.6	0.8645
OrL to horiz	22.98	0.12	23.02	0.25	0.7375
OrL to sagit	33.6	0.35	33.97	0.48	0.1549
OrL to front	15.73	0.27	15.76	0.28	0.854
OrR to horiz	22.98	0.12	23.02	0.25	0.7375
OrR to sagit	31.32	0.53	30.89	0.45	0.1591
OrR to front	18.15	0.39	18.07	0.46	0.7452

T0, before treatment; T1, after treatment, which was simulated via mandibular rearrangement; OrL, left orbitale; OrR, right orbitale; PNS, posterior nasal spine; horiz, horizontal plane; sagit, sagittal plane; front, frontal plane.

**Table 4:** Linear changes in mandibular dental landmarks among T0, T1 and T2.

	T0		T1		T2			*p*-Value	
	Mean (mm)	SD	Mean (mm)	SD	Mean (mm)	SD	T0 vs. T1	T1 vs. T2	T0 vs. T2
L7 to horiz	22.09	0.23	22.27	0.16	22.17	0.18	0.291	0.664	0.774
L7 to sagit	26.93	0.32	28.35	0.29	27	0.2	0.000 ***	0.000 ***	0.888
L7 to front	36.62	0.43	38.4	0.26	36.57	0.24	0.000 ***	0.000 ***	0.956
R7 to horiz	21.83	0.3	21.81	0.33	21.8	0.31	0.996	0.997	0.987
R7 to sagit	25.24	0.4	23.61	0.46	24.81	0.55	0.000 ***	0.002 **	0.293
R7 to front	41.36	0.25	42	0.34	41.25	0.34	0.008 **	0.002 **	0.803
R1 to horiz	30.54	0.19	31.08	0.15	30.63	0.23	0.001 ***	0.003 **	0.747
R1 to sagit	2.74	0.48	0.71	0.39	2.76	0.34	0.000 ***	0.000 ***	0.995
R1 to front	0.71	0.17	0.86	0.13	0.89	0.24	0.394	0.962	0.273

T0, before treatment; T1, after treatment, which was simulated via mandibular rearrangement; T2, after mandibular voxel-based superimposition on T1 CBCT data; L7, left mandibular second molar; R7, right mandibular second molar; R1, right mandibular central incisor; horiz, horizontal plane; sagit, sagittal plane; front, frontal plane; ** *p* ˂ 0.01; *** *p* ˂ 0.001.

**Table 5:** The linear changes in mandibular skeletal landmarks at T0, T1 and T2.

	T0		T1		T2			*p*-Value	
	Mean (mm)	SD	Mean (mm)	SD	Mean (mm)	SD	T0 vs. T1	T1 vs. T2	T0 vs. T2
MfL to horiz	53.15	0.33	53.06	0.41	53.03	0.2	0.888	0.987	0.812
MfL to sagit	22.35	0.28	24.1	0.48	22.36	0.43	0.000 ***	0.000 ***	0.999
MfL to front	24.75	0.4	27.12	0.16	24.76	0.37	0.000 ***	0.000 ***	0.999
MfR to horiz	51.32	0.4	51.84	0.4	51.42	0.24	0.054	0.136	0.867
MfR to sagit	21.68	0.34	20.37	0.15	21.69	0.37	0.000 ***	0.000 ***	0.999
MfR to front	28.96	0.19	30.13	0.28	29.08	0.36	0.000 ***	0.000 ***	0.76
GoL to horiz	35.31	0.76	35.42	0.26	35.6	0.35	0.932	0.818	0.608
GoL to sagit	48.99	0.45	49.88	0.25	49.19	0.31	0.001 **	0.010 **	0.604
GoL to front	74.91	0.73	76.57	0.4	74.46	0.33	0.000 ***	0.000 ***	0.315
GoR to horiz	35.04	0.55	34.74	0.48	35.26	0.72	0.651	0.296	0.792
GoR to sagit	39.48	0.55	39.12	0.51	39.24	0.6	0.519	0.924	0.746
GoR to front	83.8	0.3	84.2	0.9	83.47	0.58	0.539	0.15	0.647
Gn to horiz	66.09	0.41	66.71	0.27	66.23	0.4	0.027 *	0.087	0.805
Gn to sagit	0.61	0.28	0.91	0.44	0.74	0.44	0.426	0.748	0.848
Gn to front	18.26	0.38	20.77	0.5	18.65	0.55	0.000 ***	0.000 ***	0.362

T0, before treatment; T1, after treatment, which was simulated via mandibular rearrangement; T2, after mandibular voxel-based superimposition on T1 CBCT data; MfL, left mental foramen; MfR, right mental foramen; GoL, left gonion; GoR, right gonion; Gn, gnathion; horiz, horizontal plane; sagit, sagittal plane; front, frontal plane; * *p* ˂ 0.05; ** *p* ˂0.01; *** *p* ˂0.001.

**Table 6:** Angular changes in mandibular measurement planes at T0, T1 and T2.

	T0		T1		T2			*p*-Value	
	Mean (°)	SD	Mean (°)	SD	Mean (°)	SD	T0 vs. T1	T1 vs. T2	T0 vs. T2
OP with horiz	12.16	0.24	12.91	0.19	12.24	0.33	0.000 ***	0.001 **	0.851
OP with sagit	89.19	0.26	89.61	0.43	89.24	0.21	0.079	0.127	0.961
OP with front	77.88	0.24	77.1	0.19	77.79	0.33	0.000 ***	0.001 **	0.83
MP with horiz	26.75	0.44	27.89	0.47	26.95	0.71	0.008 **	0.026 *	0.811
MP with sagit	87.58	0.41	88.1	0.22	87.56	0.41	0.064	0.051	0.992
MP with front	63.38	0.43	62.19	0.49	63.18	0.74	0.007 **	0.022 *	0.823
OP with horiz	12.16	0.24	12.91	0.19	12.24	0.33	0.000 ***	0.001 **	0.851
OP with sagit	89.19	0.26	89.61	0.43	89.24	0.21	0.079	0.127	0.961
OP with front	77.88	0.24	77.1	0.19	77.79	0.33	0.000 ***	0.001 **	0.83
MP with horiz	26.75	0.44	27.89	0.47	26.95	0.71	0.008 **	0.026 *	0.811
MP with sagit	87.58	0.41	88.1	0.22	87.56	0.41	0.064	0.051	0.992
MP with front	63.38	0.43	62.19	0.49	63.18	0.74	0.007 **	0.022 *	0.823
OP with horiz	12.16	0.24	12.91	0.19	12.24	0.33	0.000 ***	0.001 **	0.851
OP with sagit	89.19	0.26	89.61	0.43	89.24	0.21	0.079	0.127	0.961
OP with front	77.88	0.24	77.1	0.19	77.79	0.33	0.000 ***	0.001 **	0.83

T0, before treatment; T1, after treatment, which was simulated via mandibular rearrangement; T2, after mandibular voxel-based superimposition on T1 CBCT data; OP, occlusal plane; MP, mandibular plane; horiz, horizontal plane; sagit, sagittal plane; front, frontal plane; * *p* ˂ 0.05; ** *p* ˂ 0.01; *** *p* ˂ 0.001.

## Data Availability

Data can be provided upon reasonable request from the corresponding author.
